# Assessment of heterogeneity between European Populations: a Baltic and Danish replication case-control study of SNPs from a recent European ulcerative colitis genome wide association study

**DOI:** 10.1186/1471-2350-12-139

**Published:** 2011-10-13

**Authors:** Vibeke Andersen, Anja Ernst, Jurgita Sventoraityte, Limas Kupcinskas, Bent A Jacobsen, Henrik B Krarup, Ulla Vogel, Laimas Jonaitis, Goda Denapiene, Gediminas Kiudelis, Tobias Balschun, Andre Franke

**Affiliations:** 1 Medical Department, Viborg Regional Hospital, DK-8800 Viborg, Denmark; 2 Medical Department, SHS Aabenraa, DK-6200 Aabenraa, Denmark; 3 Department of Clinical Biochemistry, Aarhus University Hospital, DK-9100 Aalborg, Denmark; 4 Department of Gastroenterology, Lithuanian University of Health Sciences, Kaunas, Lithuania; 5 Department of Medical Gastroenterology, Aarhus University Hospital, DK-9100 Aalborg, Denmark; 6 National Food Institute, Technical University of Denmark, DK-2860 Søborg, Denmark; 7 Institute for Science, Systems and Models, University of Roskilde, DK-4000 Roskilde, Denmark; 8 National Research Centre for the Working Environment, DK-2100 Copenhagen, Denmark; 9 Center of Hepatology, Gastroenterology and Dietetics, Vilnius University, Vilnius, Lithuania; 10 Institute of Clinical Molecular Biology, Christian-Albrechts University, Kiel, Germany

## Abstract

**Background:**

Differences in the genetic architecture of inflammatory bowel disease between different European countries and ethnicities have previously been reported. In the present study, we wanted to assess the role of 11 newly identified UC risk variants, derived from a recent European UC genome wide association study (GWAS) (Franke *et al*., 2010), for 1) association with UC in the Nordic countries, 2) for population heterogeneity between the Nordic countries and the rest of Europe, and, 3) eventually, to drive some of the previous findings towards overall genome-wide significance.

**Methods:**

Eleven SNPs were replicated in a Danish sample consisting of 560 UC patients and 796 controls and nine missing SNPs of the German GWAS study were successfully genotyped in the Baltic sample comprising 441 UC cases and 1156 controls. The independent replication data was then jointly analysed with the original data and systematic comparisons of the findings between ethnicities were made. Pearson's χ^2^, Breslow-Day (BD) and Cochran-Mantel-Haenszel (CMH) tests were used for association analyses and heterogeneity testing.

**Results:**

The rs5771069 (*IL17REL*) SNP was not associated with UC in the Danish panel. The rs5771069 (*IL17REL*) SNP was significantly associated with UC in the combined Baltic, Danish and Norwegian UC study sample driven by the Norwegian panel (OR = 0.89, 95% CI: 0.79-0.98, P = 0.02). No association was found between rs7809799 *(SMURF1/KPNA7) *and UC (OR = 1.20, 95% CI: 0.95-1.52, P = 0.10) or between UC and all other remaining SNPs. We had 94% chance of detecting an association for rs7809799 *(SMURF1/KPNA7) *in the combined replication sample, whereas the power were 55% or lower for the remaining SNPs.

Statistically significant P_BD _was found for OR heterogeneity between the combined Baltic, Danish, and Norwegian panel versus the combined German, British, Belgian, and Greek panel (rs7520292 (P = 0.001), rs12518307 (P = 0.007), and rs2395609 (TCP11) (P = 0.01), respectively).

No SNP reached genome-wide significance in the combined analyses of all the panels.

**Conclusions:**

This replication study supports an important role for the studied rs5771069 (*IL17REL*) SNP, but not for rs7809799 (*SMURF1*/*KPNA7*), in UC etiology in the Danish, Baltic, and Norwegian populations. Significant genetic heterogeneity was suggested for rs7520292, rs12518307, and rs2395609 (*TCP11*) in UC etiology between the Nordic and the other European populations.

## Background

The chronic inflammatory bowel diseases (IBD), ulcerative colitis (UC) and Crohn's disease (CD), are complex diseases caused by an interplay between genetic and environmental factors [1]. The recent years have brought much progress regarding the genetic etiology of IBD [1-15] and the number of confirmed IBD associated loci has risen to more than 99 confirmed associations [5,10-17], with more than 28 shared CD/UC risk loci. For a detailed review of the genetic findings and the genetic overlap with other inflammatory diseases see Lees *et al*. [18].

Studies have found that disease distribution and phenotypic appearance differ significantly between ethnic groups and even within populations [19-21] although other studies disagree [22]. Thus, uneven geographic distribution of IBD has been found within European countries such as Scotland [23] and France [24], and also within the USA [25]. Apart from varying environmental factors that affect susceptible individuals, genetic heterogeneity between different populations itself plays an important role.

Emerging evidence suggests that the contribution from each gene to IBD development may vary considerably among different populations. Heterogeneity has been found for IBD risk candidate genes such as *COX-2 *[26] and *ABCB1 *(*MDR1*) [27-29] gene variants. Also the CD-associated *NOD2 *variants have demonstrated a remarkable amount of heterogeneity across ethnicities and populations [30,31]. Case control studies have found that the contribution to IBD disease susceptibility of the *NOD2 *variants is low in the non-Caucasian population [32-34] and, furthermore, in the Swedish, Norwegian, Baltic, and Danish populations [12,35-41]. In spite of this, a meta-analyses found no difference between the impact of the CD-associated *NOD*2 variants in the Northern and Southern European populations, which may be due to the methodological reason as e.g. the German panel was included in the Northern European populations [42]. Thus, the Nordic populations may have a distinct pattern of IBD risk-associated gene variants compared to Central European countries. It is therefore of particular interest to search for genetic determinants in these populations which are characterised by few identified susceptibility genes.

In the present study, we wanted to assess the role of 11 newly identified UC risk variants, derived from a recent European UC genome wide association study (GWAS), for association with UC in the Nordic countries [7]. In the specific GWAS, Franke and colleagues identified 11 SNPs which were subsequently replicated in an independent sample set [7]. Furthermore, we wanted to evaluate these 11 SNPs for population heterogeneity between the Nordic countries and the rest of Europe, and, eventually, we wanted to drive the previous findings towards overall genome-wide significance. We genotyped all 11 SNPs in a Danish UC case-control sample and 9 SNPs, which were previously not tested in the respective panel, were genotyped in a Baltic UC case-control sample. Furthermore, we included the previously published data on the 2 SNPs, rs7809799 and rs5771069, from the Baltic sample and on all 11 SNPs from the Norwegian, Belgian, British, German and Greek sample from the primary GWAS study in the analyses [7]. In total, we analysed 3097 cases and 6223 controls from the Baltic, Danish and Norwegian samples (1275 cases and 2234 controls) and the Belgian, British, German and Greek panels (1822 cases and 3989 controls).

## Methods

### Patients and controls

The Baltic study population consisted of 441 UC patients and 1156 controls recruited at 6 hospitals in Lithuania (401 cases and 1099 controls) and 2 hospitals in Latvia (40 cases and 57 controls) [7]. The Danish study population consisted of 560 UC patients and 796 healthy blood donors recruited at 3 hospitals in the Northern part of Jutland [15]. All study participants were of Caucasian ethnicity based on self-reported information. Diagnosis of UC was based on standard clinical, radiological, endoscopic and histological criteria [43].

### Selection of single nucleotide polymorphisms

The 11 SNPs of the formerly published GWAS were selected because they were reported as robust replications [7] leaving out SNPs that were found to have significantly different allele frequencies when comparing the different countries (P_BC _< 0.05; P-value for Breslow day test), or which were previously replicated as UC-associated variants [7]. Thus, 11 SNPs were selected: rs7520292, rs1488266 (*BTLA*), rs3103190, rs12518307, rs2395609 (*TCP11*), rs7809799 *(SMURF1/KPNA7)*, rs6966125 (*TNPO3*), rs638300, rs6119625 (*FER1L4*), rs6125345, and rs5771069 (*IL17REL*). Genotype data on all 11 SNPs in the Norwegian, Belgian, British, German and Greek panels was derived from the original GWAS replication study and included in further analyses. Rs5771069 and rs7809799 in the Baltic panel had been genotyped in the origininal GWAS replication study. The depletion of DNA stocks required an exchange of some samples used in the original analysis, however, the sampleset used for this analysis here is >98% concordant to the one used in the Franke et al paper. For not introducing a bias by using experimental data for the 2 previously typed SNPs we also typed these 2 SNPs again. Therefore, 11 SNPs were genotyped in the Danish and Baltic cohort.

### Genotyping

Functionally tested TaqMan^® ^SNP Genotyping Assays (Applied Biosystems, Foster City, USA) were used to genotype the 11 SNPs in the Danish sampleset and the 9 SNPs missing in the Baltic [44]. SNPlex were used for the Belgian, British, German and Greek panels and the Affymetrix Genome-Wide Human SNP Array 5.0 for the Norwegian sample set as previously described [7]. All process data were written to and administered by a previously described database-driven laboratory information management system (LIMS) [45]. Duplicate or related samples were identified and excluded from the analyses, using algorithms implemented in the LIMS. However, no specific genotyping was conducted to "ID" tag the DNA samples. HapMap controls with known genotypes were included in each run, and repeated genotyping of a random 10% subset yielded 100% identical genotypes. Genotypes were obtained for less than 95% of the Danish study group only for SNP rs6125345 and excluded from further analyses. The genotype distributions among the controls did not deviate from Hardy-Weinberg equilibrium (P_HWE _< 0.05) in either of the study populations.

### Association analyses

Genotype data from the present study and from pre-existing GWAS on rs5771069 and rs7809799 from the Baltic cohort and on all the 11 SNPs from the Norwegian, Belgian, British, German and Greek cohorts [7] were analysed using gPLINK v2.050 in combination with PLINK v1.07 [46]. Pearson's χ^2 ^test on allele counts (P_CCA_; 1 degree of freedom) was used for the separate analyses of the Baltic and Danish cohorts. The asymptomatic P-value of the Breslow-Day (P_BD_) test was used for analysing the heterogeneity of the odds ratios (ORs). The Cochran-Mantel-Haenszel (CMH) test was used for statistic analyses of the combined P-values (P_CMH_) and combined odds ratios. We used the tool "Confidence Interval of differences and Forest Plots" and Microsoft Office Excel 2007 for forest plot analyses [47].

### Power analyses

We used the tool "Genetic Power Calculator" [46] for power analyses. The minor allele frequencies in the control group and ORs were retrieved from Franke et al [7] and used for "high risk allele frequency" and "genotype relative risk", respectively, the "prevalence" was set to 0.001, D-prime was set to 1, number of cases was 1275 and control:case ratio 0.9. Type I error rate = 0.05, user-defined power (1-type II error rate) = 0.80.

### Ethical Considerations

The study was conducted in accordance with the Declaration of Helsinki. Written, informed consent was obtained from all study participants. All protocols were approved by the national and institutional ethical review committees of the participating centres (Lithuania 2/2008, Latvia 290910-8L) or local Scientific Ethical Committees (Denmark) (VN 2003/5 and VN 2003/124).

## Results

Detailed characteristics of the Baltic, Danish and Norwegian IBD patients and controls are shown in Table 1. A total of 1275 cases and 2234 controls were included in the analyses.

**Table 1:** Participant description

	Baltic		Danish		Norwegian	
	cases	controls	cases	controls	cases	Controls
	N = 441	N = 1156	N = 560	N = 796	N = 274	N = 282
Sex distribution						
% female	50	51	48	52	48	41
Age at sampling						
Median age (years, SD)	44 [17]	40 [12]	49 [16]	43 [11]	39 [15]	32 [7]
Age of diagnosis						
Median age (years, SD)	38 [16]		35 [11]		38 [15]	
Disease extent, %						
Left sided colitis	76		80		69	
Extensive colitis	24		20		31	
Smoking habits, %						
Current	12		17		13	
previous	28		36		31	
never	60		47		56	

In all three panels age- and sex-matched controls have been used.

### Analyses for SNP associations in the separate Baltic, Danish, and Norwegian case-control panels

Table 2 shows the allele frequencies, association results and ORs of the 11 SNPs for the separate Baltic, Danish, and Norwegian case-control panels. A P-value of less than 0.05 was considered as statistically significant as all variants are independent from each other and previously described as true associations, i.e. no correction for multiple testing was applied. Rs12518307 was associated with risk of UC in the Danish cohort at the 5% level (p < 0.05). As previously reported by Franke et al. (table S-five) [7] rs7809799 was associated with risk of UC in the Baltic cohort, and rs5771069 with risk of UC in the Norwegian cohort (*IL17REL*). The allele frequencies for the 11 SNPs in the combined German, British, Belgian, and Greek panel are shown for comparison reasons (Table 2).

**Table 2:** Odds ratios (OR) for the 11 SNPs for the Baltic, Danish and Norwegian UC panels^1^

		Baltic^1^ 441 UC cases/1156 controls					Danish 560 UC cases/796 controls					Norwegian^1^ 274 UC cases/282 controls					European^1^ 3989 co/1822 ca
**SNP**	**A1**	**F_CA_**	**F_CO_**	**OR**	**95% CI**	**P_**CCA**_**	**F_**CA**_**	**F_**CO**_**	**OR**	**95% CI**	**P_**CCA**_**	**F_**CA**_**	**F_**CO**_**	**OR**	**95% CI**	**P_**CCA**_**	**F_**CA**_**

rs7520292	A	0.20	0.20	1.04	0.85-1.25	0.71	0.17	0.16	1.01	0.81-1.24	0.94	0.16	0.16	1.00	0.73-1.38	0.96	0.20
rs1488266 *BTLA*	C	0.06	0.06	0.95	0.69-1.29	0.72	0.10	0.11	0.91	0.71-1.16	0.45	0.09	0.10	0.90	0.60-1.36	0.64	0.10
rs3103190	G	0.40	0.41	0.98	0.83-1.14	0.76	0.30	0.33	0.87	0.73-1.02	0.09	0.31	0.31	0.99	0.77-1.27	0.95	0.34
rs12518307	A	0.33	0.33	1.00	0.84-1.17	0.99	0.27	0.23	1.19	1.00-1.42	**0.04**	0.27	0.26	1.04	0.79-1.35	0.77	0.25
rs2395609 *TCP11*	G	0.29	0.30	0.92	0.77-1.09	0.35	0.34	0.33	1.04	0.88-1.23	0.60	0.35	0.36	0.97	0.76-1.24	0.83	0.32
rs7809799 *SMURF1/KPNA7*1	G	0.07	0.05	1.42	1.03-1.94	**0.03**	0.03	0.04	0.86	0.57-1.27	0.44	0.04	0.02	1.6	0.86-3.23	0.11	0.05
rs6966125 *TNP03*	G	0.21	0.20	1.01	0.83-1.22	0.89	0.15	0.14	1.10	0.88-1.36	0.40	0.12	0.14	0.84	0.59-1.19	0.33	0.16
rs638300	T	0.15	0.14	1.09	0.88-1.35	0.42	0.20	0.20	0.97	0.80-1.17	0.77	0.21	0.19	1.10	0.82-1.48	0.49	0.21
rs6119625 *FER1L4*	G	0.15	0.14	1.08	0.87-1.34	0.47	0.13	0.12	1.11	0.88-1.39	0.37	0.13	0.10	1.28	0.89-1.83	0.17	0.15
rs6125345	C	0.17	0.18	0.93	0.75-1.13	0.45						0.19	0.16	1.20	0.88-1.63	0.24	0.20
rs5771069 *IL17REL*	A	0.50	0.52	0.93	0.79-1.08	0.31	0.49	0.50	0.97	0.82-1.12	0.67	0.43	0.53	0.69	0.54-0.87	**0.002**	0.49

^1^Data for the 11 SNPs in the Norwegian panel, for rs5771069 and rs7809799 in the Baltic panel, and the allele frequencies for the combined German, British, Belgian, and Greek panel is from Franke *et al *[7].

A1: minor allele; F: allele frequency of A1 for cases (F_CA_) or controls (F_CO_), respectively; 95%CI: 95% confidence interval; P_CCA_: P-value (Pearson's χ^2 ^test on allele counts with one degree of freedom); nominal significant P-values are highlighted by bold type

### Analyses for SNP associations in the combined Baltic, Danish, and Norwegian panel

**Figure 1:**
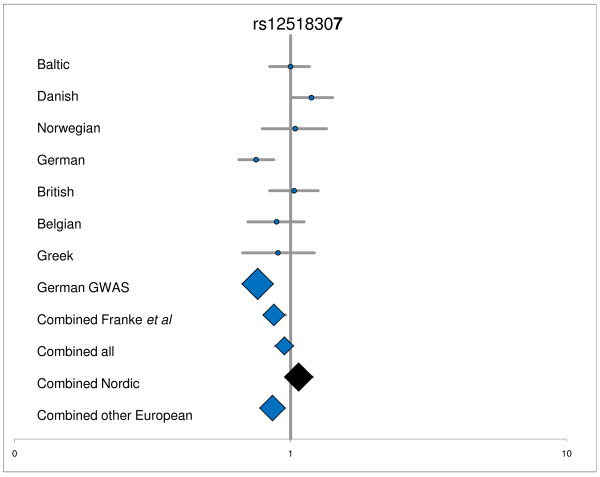
**Detailed analyses of rs12518307**. ^1^Data for for Norwegian, German, British, Belgian, and Greek panel, and the original German GWAS is from Franke *et al *[7]; Combined Franke *et al *= Norwegian, German, British, Belgian, and Greek panel; Combined all = Baltic, Danish, Norwegian, German, British, Belgian, and Greek panel; Combined Nordic = Baltic, Danish, and Norwegian panel; Combined other European = German, British, Belgian, and Greek panel; nominal significant P-values are highlighted by blue.

**Figure 2:**
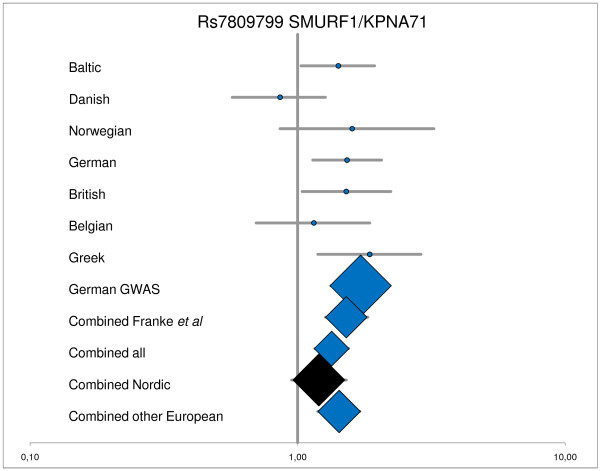
**Detailed analyses of rs7809799 SMURF1/KPNA71**. ^1^Data for rs7809799 in the Baltic panel, and for Norwegian, German, British, Belgian, and Greek panel, and the original German GWAS is from Franke *et al *[7]; Combined Franke *et al *= Norwegian, German, British, Belgian, and Greek panel; Combined all = Baltic, Danish, Norwegian, German, British, Belgian, and Greek panel; Combined Nordic = Baltic, Danish, and Norwegian panel; Combined other European = German, British, Belgian, and Greek panel; nominal significant P-values are highlighted by blue.

Table 3 shows OR for the 11 SNPs and risk of UC for the combined Baltic, Danish and Norwegian panel versus the combined German, British, Belgian, and Greek panel. Figure 1 and 2 shows the OR for rs7809799 *(SMURF1/KPNA7) *and rs5771069 (*IL17REL*) for the individual panels and combined panels, respectively. The rs5771069 (*IL17REL*) SNP is statistically significantly associated with UC in the combined Northern European sample set (OR = 0.89, 95% CI: 0.79-0.98, P = 0.02). Figure 2 and the P_CCA _values show that this association was mainly based on the Norwegian panel as has previously been reported by Franke et al [7]. In the study by Franke et al [7] the rs5771069 G allele is denoted as the A1 allele, whereas in this study the rs5771069 A allele is denoted as the A1 allele. No association between rs7809799 *(SMURF1/KPNA7) *and UC was observed (OR = 1.20, 95% CI: 0.95-1.52, P = 0.10; the *a priori *estimated chance of detection was 94%). No other association between any of the studied SNPs and UC was identified in the combined Northern European panel. The estimated power of the combined Baltic, Danish and Norwegian panel to detect a possible association at the α = 0.05 significance level, assuming a variant allele frequency similar to what was observed in the study by Franke *et al*. [7] (only data from replication panel used, i.e. inflated GWAS data excluded), is shown in Table 3. The power to detect a real association was 94% for the rs7809799 *(SMURF1/KPNA7)*, whereas the power was 55% or lower for the remaining SNPs.

**Table 3:** Odds ratios (OR) for the combined UC panels and heterogeneity test for the Northern countries (Baltic, Norwegian, and Danish) versus rest of Europe (German, British, Belgian, and Greek).

		Baltic, Norwegian, and Danish^1^ 1275 cases and 2234 controls					German, British, Belgian, and Greek^1^ 1822 cases and 3989 controls				Northern vs rest of Europe	Baltic, Norwegian, Danish, German, British, Belgian, and Greek 3097 cases/6223 controls		
**SNP**	**A1**	**P_BD_**	**OR_CMH_**	**95%CI**	**P_CMH_**	**Power**	**P_BD_**	**OR_CMH_**	**95%CI**	**P_CMH_**	**P_BD_**	**OR_CMH_**	**95%CI**	**P_CMH_**

rs7520292	A	0.98	1.02	0.89-1.16	0.75	43%	0.13	1.19	1.08-1.32	**0.004**	**0.001**	1.13	1.04-1.22	**0.003**
rs1488266 *BTLA*	C	0.98	0.92	0.77-1.09	0.35	49%	0.50	0.83	0.72-0.95	**0.01**	0.14	0.87	0.78-0.97	**0.01**
rs3103190	G	0.54	0.93	0.84-1.03	0.20	30%	0.06	0.86	0.79-0.94	**0.001**	0.71	0.89	0.84-0.96	**0.001**
rs12518307	A	0.34	1.07	0.96-1.20	0.18	39%	0.17	0.86	0.78-0.95	**0.002**	**0.007**	0.95	0.88-1.02	**0.16**
rs2395609 *TCP11*	G	0.59	0.98	0.88-1.09	0.73	39%	0.17	0.84	0.77-0.92	**0.0002**	**0.01**	0.90	0.84-0.96	**0.002**
rs7809799 *SMURF1/KPNA7*1	G	0.09	1.20	0.95-1.52	0.10	94%	0.81	1.43	1.19-1.71	**0.0001**	0.10	1.34	1.16-1.55	**0.00005**
rs6966125 *TNP03*	G	0.45	1.01	0.88-1.16	0.82	54%	0.34	0.83	0.73-0.93	**0.001**	0.12	0.90	0.83-0.99	**0.02**
rs638300	T	0.65	1.03	0.91-1.18	0.55	55%	0.10	1.19	1.08-1.31	**0.0003**	0.18	1.14	1.05-1.23	**0.001**
rs6119625 *FER1L4*	G	0.72	1.12	0.97-1.29	0.11	47%	0.90	1.19	1.06-1.33	**0.001**	0.29	1.17	1.07-1.28	**0.0006**
rs6125345	C	0.33	1.03	0.90-1.18	0.64	39%	0.45	1.15	1.03-1.27	**0.006**	0.29	1.11	1.02-1.20	**0.01**
rs5771069 *IL17REL*	A	0.06	0.89	0.79-0.98	**0.02**	29%	0.54	0.89	0.82-0.97	**0.008**	0.86	0.89	0.84-0.95	**0.0005**

^1^Data on the 11 SNPs in the Norwegian, German, British, Belgian, and Greek cohort, and on rs5771069 and rs7809799 in the Baltic cohort is derived from the study of Franke *et al *[7]. In the study by Franke et al [7] the rs5771069 G allele is denoted as the A1 allele, whereas in this study the rs5771069 A allele is denoted as the A1 allele. Combined P-values (P_CMH_) and combined ORs (OR_CMH_) of the Cochran-Mantel-Haenszel (CMH) test statistic; Breslow-Day test for odds ratio heterogeneity between countries (P_BD_); A1: minor allele in healthy controls; F: allele frequency of A1 for cases (F_CA_) and controls (F_CO_), respectively; 95%CI: 95% confidence interval; nominal significant P-values are highlighted by bold type;

Breslow-Day significance tests for odds ratio heterogeneity (P_BD_) between the panels in the combined analyses did not reach statistical significance for any of the SNPs (Table 3).

### Analyses for heterogeneity between the combined Baltic, Danish, and Norwegian panel and the combined German, British, Belgian, and Greek panel

**Figure 3:**
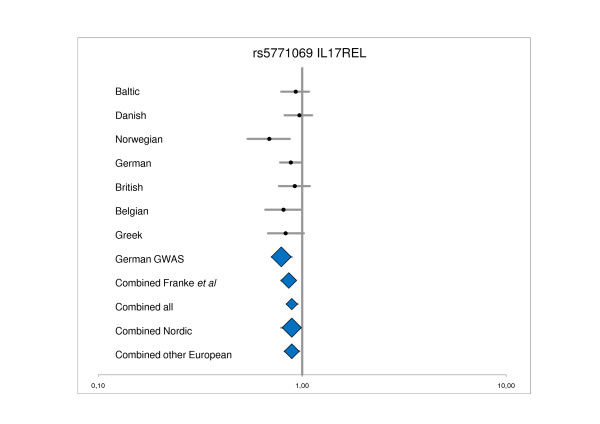
**Detailed analyses of and rs5771069 IL17REL**. ^1^Data for rs5771069 in the Baltic panel, and for Norwegian, German, British, Belgian, and Greek panel, and the original German GWAS is from Franke *et al *[7]; Combined Franke *et al *= Norwegian, German, British, Belgian, and Greek panel; Combined all = Baltic, Danish, Norwegian, German, British, Belgian, and Greek panel; Combined Nordic = Baltic, Danish, and Norwegian panel; Combined other European = German, British, Belgian, and Greek panel; nominal significant P-values are highlighted by blue.

The result of the analyses for OR heterogeneity between the combined Baltic, Danish, and Norwegian panel versus the combined German, British, Belgian, and Greek panel is shown in Table 3. Statistically significant P_BD _was found for 3 SNPs; the rs7520292 (P = 0.001), rs12518307 (P = 0.007), and rs2395609 (*TCP11*) (P = 0.01), respectively. For comparison, table 3 shows the re-analysed results of the replication panel from the original GWAS study including data from the other European panels; i.e. the German, British, Belgian, and Greek sample sets (1822 cases and 3989 controls) (previously published in Supplementary Table S-four in [7]). Figure 3 shows the OR for rs12518307 for the individual panels and combined panels.

### Analyses for SNP associations in the combined Baltic, Danish, Norwegian, German, British, Belgian, and Greek panel

Table 3 shows OR for the 11 SNPs and risk of UC for the combined Baltic, Danish, Norwegian, German, British, Belgian, and Greek panel, including 3097 cases and 6223 controls. No association between any of the studied SNPs and UC on genome-wide significance level (P < 5 × 10^-8) ^was identified in the combined panel.

## Discussion

The present case-control study assessed the association with UC in Northern European panels, comprising 1275 patients and 2234 controls from Denmark, Norway, Lithuania and Latvia, for 11 recently identified SNPs [7]. In the combined analyses of the three panels, rs5771069 (*IL17REL*), but not rs7809799 (*SMURF1*/*KPNA7*), was statistically significantly associated with UC. Statistically significant heterogeneity between the combined panel from the three Nordic countries versus the other European panels was found for three SNPs (rs7520292, rs12518307, and rs2395609 (*TCP11*)). No SNP reached genome-wide significance in the combined analyses of all the panels.

Although this study found significant association in the combined Northern European panels between UC and rs5771069 (*IL17REL*), rs5771069 did not show association in the Baltic (data from [7]) or Danish panel. Thus, this result was based on the Norwegian sample (data from [7]). Next, we were not able to replicate the association with rs7809799 (*SMURF1/KPNA71*) in spite of sufficient power which suggests that this SNP does not contribute to UC etiology in these countries. The associations found in the separate analyses of the three Northern European sample sets may be due to chance and therefore needs replication in other samples to be verified. It is important to stress the strengths and limitations of our study. We treat each association test as a single, independent hypothesis test, allowing for a nominal significance threshold. The associations, in general, did not withstand Bonferroni correction for multiple testing with a significant P-value of 0.05/11 SNP tested = 0.0045. However, Bonferroni correction may be overly conservative in our setting where we aimed to replicate previously identified risk variants [48]. Although this study had 94% power to detect a real association between rs7809799 (*SMURF1/KPNA71*) and UC in the combined Baltic, Danish, and Norwegian study sample if there was one, the power to detect an association with e.g. rs5771069 (*IL17REL*) was low. Therefore, further recruitment efforts are essential to increase the Northern European study sample size for validating the importance of the yet established susceptibility loci.

Next, our study suggests genetic heterogeneity between the Northern and the rest of the European populations. The Breslow-Day test for odds ratio heterogeneity was significant for rs7520292, rs12518307, and rs2395609 (*TCP11*) between the combined Baltic, Danish, and Norwegian sample and the combined German, British, Belgian, and Greek sample (Table 3). Interestingly, also within the Northern countries 2 SNPs pointed to a certain amount of population stratification. Although not statistically significant, the Breslow-Day test found values of 0.06 and 0.09 for rs7809799 (*SMURF1/KPNA71*) and rs5771069 (*IL17REL*), respectively. Varying environmental exposure between countries for factors that affect susceptible individuals may contribute to these findings, i.e. environmental exposures such as tobacco smoking and diet [49,50] may alter the effects of possessing certain risk genes and next the risk of IBD. Thereby, differences in environmental risk factors between countries may contribute to differences in the identified risk genes. Thus, genetic heterogeneity between the different populations in combination with varying environmental exposure impedes the detection of real associations, preferring carefully selected combinations of different panels. Investigating the detailed structure of the Baltic countries and other North-Eastern European populations, revealed that the Baltic countries Poland and Western Russia together form a genetic cluster [51], thereby indicating that the two Baltic study populations can be combined in association analyses. Eventually, population-based genotyping and estimation of the penetrance of disease susceptibility genes in different populations will teach us about the impact of the genetic heterogeneity [36].

Finally, no SNP were found to be associated with UC at a genome-wide level in the analyses of all panels combined.

In summary, our study suggest that rs5771069 (*IL17REL*) is a genetic susceptibility factor in Northern European countries [7], whereas we failed to replicate the association of rs7809799 with UC in the present adequately-powered study. The data suggest significant heterogeneity for rs7520292, rs12518307, and rs2395609 (*TCP11*) between the Northern and the rest of the European populations. Eventually, we were not able to reach genome-wide association level of any of the investigated SNPs with UC.

## Conclusions

This study supports an important role for the studied rs5771069 (*IL17REL*) SNP, but not for rs7809799 (*SMURF1*/*KPNA7*), in UC etiology in the Northern European population. Significant genetic heterogeneity was suggested for rs7520292, rs12518307, and rs2395609 (*TCP11*) in UC etiology between the Northern and the rest of the European populations.

## Competing interests

The authors declare that they have no competing interests.

## Authors' contributions

TB and AE carried out the genotyping. VA, HK, AE, JS, and LK established the patient panels and/or participated in sample preparation and collection. TB performed the statistical analyses. AF, TB, VA and UV conceived the genotyping study, and its design and coordination and VA wrote the manuscript draft. All authors read, edited and approved the final manuscript.

## Pre-publication history

The pre-publication history for this paper can be accessed here:

http://www.biomedcentral.com/1471-2350/12/139/prepub
